# An eIF4E-interacting peptide induces cell death in cancer cell lines

**DOI:** 10.1038/cddis.2014.457

**Published:** 2014-10-30

**Authors:** M Masse, V Glippa, H Saad, R Le Bloas, I Gauffeny, C Berthou, M Czjzek, P Cormier, B Cosson

**Affiliations:** 1 Sorbonne Universités, UPMC Univ Paris 06, UMR 8227, Integrative Biology of Marine Models, Translation Cell Cycle and Development, Station Biologique de Roscoff, CS 90074, Roscoff cedex, France; 2 CNRS, UMR 8227, Integrative Biology of Marine Models, Station Biologique de Roscoff, CS 90074, Roscoff cedex, France; 3 Université Européenne de Bretagne, Brittany, France; 4 Institut de Cancéro-hématologie, Département d'Hématologie, Hôpital Morvan, CHRU, Brest, France; 5 Sorbonne Universités, UPMC Univ Paris 06, UMR 8227, Integrative Biology of Marine Models, Végétaux marins et biomolécules, Station Biologique de Roscoff, CS 90074, Roscoff cedex, France; 6 CNRS, UMR 8227, Integrative Biology of Marine Models, Végétaux marins et biomolécules, Station Biologique de Roscoff, CS 90074, Roscoff cedex, France

## Abstract

The eukaryotic initiation factor eIF4E is essential for cap-dependent initiation of translation in eukaryotes. Abnormal regulation of eIF4E has been implicated in oncogenic transformation. We developed an eIF4E-binding peptide derived from Angel1, a partner of eIF4E that we recently identified. We show here that this peptide fused to a penetratin motif causes drastic and rapid cell death in several epithelial cancer cell lines. This necrotic cell death was characterized by a drop in ATP levels with F-actin network injury being a key step in extensive plasma membrane blebbing and membrane permeabilization. This synthetic eIF4E-binding peptide provides a candidate pharmacophore for a promising new cancer therapy strategy.

The eukaryotic initiation factor eIF4E is essential for cap-dependent initiation of translation in eukaryotes and is considered to be the rate-limiting factor of protein synthesis.^1^ eIF4E binds to the 5' cap structure found on all nuclear-encoded mRNAs and to the scaffolding protein eIF4G, which along with eIF3, bridges mRNA to ribosomes.^2^ eIF4E and eIF4G form the eIF4F complex with eIF4A, an ATP-dependent RNA helicase that facilitates ribosomal scanning from the 5' cap by unwinding secondary structures within the 5' untranslated region (5'UTR). eIF4E also has a function in gene expression unrelated to translation initiation. It regulates the export of specific mRNAs, including cyclin D1, from the nucleus to the cytoplasm. ^3, 4^

eIF4E is regulated at multiple levels, including through interactions with a family of eIF4E-binding proteins that compete with eIF4G to bind to the dorsal face of eIF4E. Hypophosphorylated eIF4E binding protein 4E-BP1, the best-characterized inhibitor of eIF4E activity, sequesters eIF4E and prevents the recruitment of eIF4G to the 5' cap of mRNAs. Upon mitogen activation or stimulation with growth factors or cytokines, 4E-BP1 is phosphorylated at multiple sites by the mammalian target of rapamycin (mTOR) signaling pathway leading to its dissociation from eIF4E.^5^ Accordingly, eIF4E activity has been linked to growth stimulation and oncogenic transformation that enhance the translation of a subset of mRNAs believed to be poorly expressed in normal cellular conditions. These mRNAs predominantly encode growth factors and proto-oncogenes involved in cell proliferation and promote tumor cell survival, angiogenesis, transformation, invasion and metastasis.^6^

Cancer cells frequently show elevated levels of eIF4E,^7^ reduced expression of 4E-BP1 and activation of signaling pathways that phosphorylate 4E-BP1.^8^ Elevated levels of eIF4E are sufficient to induce deregulated growth and malignant transformation of a variety of cultured cell lines.^9^ Correlatively, overexpression of 4E-BP has been reported to partially reverse transformation mediated by the oncogenic gene v-src.^10^ Targeting eIF4E–eIF4G interactions is a potential way to reverse the aberrant activation of eIF4E in cancer.^11^ The small molecule inhibitor 4EGI-1 and an eIF4E-binding peptide were described previously to inhibit growth and to have proapoptotic activities.^12, 13^ We previously identified Angel1 as a new partner of eIF4E and we showed that Angel1 efficiently competes with eIF4G to bind to eIF4E.^14^ In the present paper, we generated a new eIF4E-interacting peptide designed from the eIF4E-binding motif of Angel1 to target eIF4E–eIF4G interactions. We demonstrate that this peptide can efficiently inhibit *in vitro* translation. Surprisingly, it also induces rapid cell death in a wide variety of cancer cell lines involving a dramatic disorganization of the F-actin network, cell blebbing and plasma membrane rupture.

## Results

### Generation of eIF4E-interacting peptides

**Figure 1:**
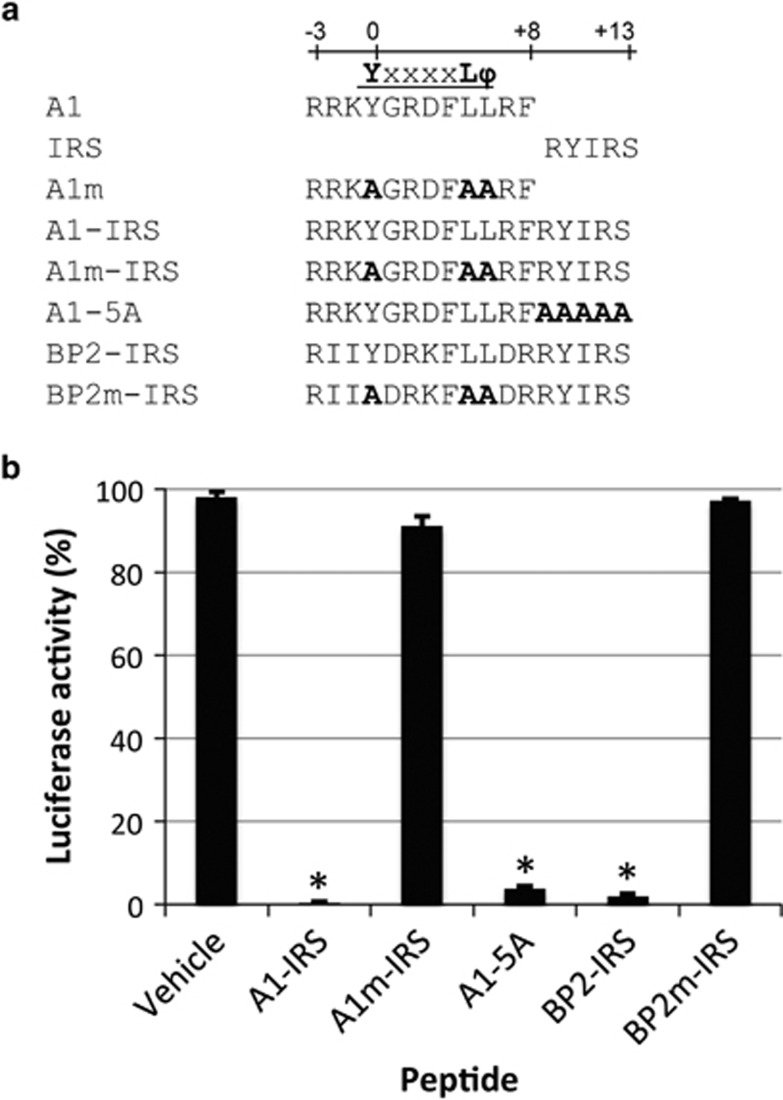
A1-IRS peptide inhibits *in vitro* translation. (**a**) Sequences of the eIF4E-binding motif of Angel1 (A1), the eIF4E-binding protein 4E-BP2 (BP2), the penetratin IRS domain (IRS) and the synthesized peptides (A1-IRS, A1m-IRS, A1-5 A, BP2-IRS, BP2m-IRS). The consensus eIF4E-binding motif YxxxxL*ϕ* is indicated. (**b**) Capped and polyadenylated Renilla luciferase mRNA was translated in rabbit reticulocyte lysate in the presence of 50 *μ*M of the indicated peptides. Renilla luciferase activity was then determined as described in Materials and Methods. Translation with the vehicle (DMSO) was arbitrarily set to 100%. The results presented are representative of three independent experiments performed in triplicates and expressed as mean±S.D. *Versus* group control (vehicle): * *P*<0.001

We recently characterized a new eIF4E-interacting partner, Angel1.^14^ The interaction site of Angel1 (designated A1) contains the consensus Y-X-X-X-X-L-ф recognition motif (where X is variable and ф is an hydrophobic residue, usually L, M or F) conserved in the 4E-BP and eIF4G families throughout evolution and described to be essential for their binding to eIF4E^15, 16^ (for Angel1, see Supplementary Figure S3 in Gosselin *et al.*^14^). We thus used this sequence to generate a new eIF4E-binding peptide. An 11 amino acid sequence derived from the recognition motif of Angel1 (from −3 to +8, annotated from the conserved tyrosine of the consensus motif, Figure 1a) was fused to the yellow fluorescent protein (YFP) as a carrier. This fusion protein, named YFP-A1 (for YFP-Angel1 peptide), was clearly retained on an eIF4E column, but not YFP alone (Supplementary Figure S1, compare lanes 5 and 2). Mutations affecting the hydrophobic amino acids tyrosine and leucine greatly decreased binding to eIF4E (Supplementary Figure S1, lane 8), an expected result because these mutations in the eIF4E-binding site of 4E-BP1 preclude binding to eIF4E in chordates.^16, 17^

A1 and the eIF4E-binding domain of 4E-BP2 (designated BP2 herein) were fused to the IRS-penetratin peptide domain^18^ (giving rise to A1-IRS and BP2-IRS, respectively, Figure 1a) to allow internalization in cultured cells (see below). An eIF4E-interacting peptide derived from 4E-BP has been shown to inhibit translation initiation;^19^ we therefore tested whether A1-IRS is also able to inhibit protein synthesis in an *in vitro* translation system. A1-IRS was as efficient as BP2-IRS in drastically inhibiting translation (Figure 1b), whereas, as expected, the consensus motif mutants did not affect translation activity (A1m-IRS and BP2m-IRS, Figure 1b). The A1-5A variant, obtained by replacing the IRS sequence with alanines, still inhibited translation activity (A1-5A, Figure 1b), indicating that the IRS-penetratin sequence had no role in translation inhibition. We validated by ^35^S Methionine incorporation that A1-IRS inhibits translation in living cells (Supplementary Figure. S2). We concluded that A1-IRS is a *bona fide* eIF4E-inhibiting peptide.

### A1-IRS induces cell blebbing that leads to cell death

**Figure 2:**
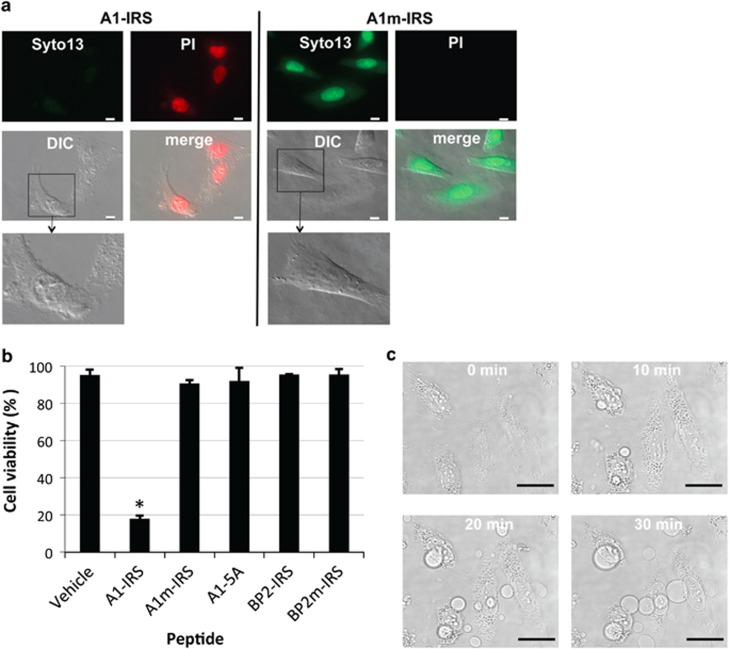
A1-IRS induces membrane blebbing and HeLa cell death as evaluated by plasma membrane permeability. HeLa cells were incubated with 50 *μ*M of the indicated synthesized peptides or with the vehicle (DMSO) control. (**a**) HeLa cells were incubated 30 min with A1-IRS (left panel) or A1m-IRS (right panel), in presence of 5 mM Syto 13 and 12.5 *μ*g/ml propidium iodide (PI). Images were collected on a Zeiss observer Z1 Fluorescence microscope using a × 63 objective. Scale bar=25 *μ*m. (**b**) Cell viability was determined as the percentage of PI-negative and Syto 13-positive cells using the ImageJ program. At least 200 cells per well were counted. The data are given as the mean±S.D. of triplicates, and are representative of three experiments. *Versus* group control (vehicle): * *P*<0.001. (**c**) A field of HeLa cells were imaged under phase contrast every 10 min using a Leica SP5 microscope with a × 63 objective. The recording begins 2 min after adding A1-IRS in the cell culture medium. Scale bar=25 *μ*m

We fused the A1 peptide to the IRS sequence to allow cellular uptake. The synthetic fusion peptide A1-IRS was efficiently taken up by HeLa cells (Supplementary Figure S3). HeLa cells loaded with the membrane-permeant green fluorescent Syto 13 dye were exposed to A1-IRS in the presence of the membrane-impermeant dye propidium iodide (PI).^20^ Cell fluorescence was monitored by microscope (Figure 2a), and cell viability was quantified (Figure 2b). Surprisingly, after a 30 min incubation with A1-IRS, most cells had incorporated the red fluorescent dye (Figures 2a and b). The PI staining was characteristic of membrane permeability and cell death. As expected, we observed loss of membrane integrity upon prolonged incubation with A1-IRS (data not shown). Importantly, cells incubated with the peptide variant unable to bind to eIF4E (A1m-IRS) were not permeable to PI and retained the green fluorescence (Figures 2a and b), as observed for the control, dimethyl sulfoxide (DMSO)-treated cells (vehicle, Figure 2b). As expected, the variant without the IRS-penetratin sequence had no effect on cell viability (A1-5A, Figure 2b). These results indicate that the peptide must contain a functional eIF4E-binding motif to induce cell death. However, incubation with BP2-IRS did not induce cell death (Figure 2b). Therefore, the specific sequence of the Angel1 eIF4E-binding motif is necessary to induce cell death.

Under differential interference contrast (DIC) microscopy, cells showed peculiar morphology after incubation with A1-IRS (Figure 2a). DIC illumination gives a pseudo three-dimensional appearance to the specimen that does not reflect the real cell shape, but nonetheless indicates regions of different thicknesses and/or refractive index. After treatment with A1-IRS, the nucleus was delineated by a ring-like structure that we assumed to be the nuclear envelope, given the PI staining (Figure 2a). The nucleus contained spherical granules that probably correspond to nucleoli. To further explore these morphological changes, we performed a time-lapse experiment. As shown in Figure 2c, incubation with A1-IRS induced massive cell blebbing, with the diameter of the blebs reaching a diameter similar to that of the nucleus. Assuming that the total cell volume is conserved, the volume and the thickness of the cell decrease with bleb growth. Consequently, cell blebbing may induce differences in the refractive index of the cell structures, explaining the specific DIC pattern observed after incubation with A1-IRS (Figure 2a).

To examine the relationship between bleb growth and cell permeabilization, both were simultaneously recorded in a HeLa cell. Blebs first appeared and reached a substantial size before cell permeabilization was observed with PI staining of the nucleus (Supplementary Movie 1). Blebs did not continue to increase after membrane permeabilization. In summary, we observed that the A1-IRS peptide caused massive cell blebbing that precedes membrane permeabilization and cell death.

### A1-IRS causes cell death in various cell lines

**Figure 3:**
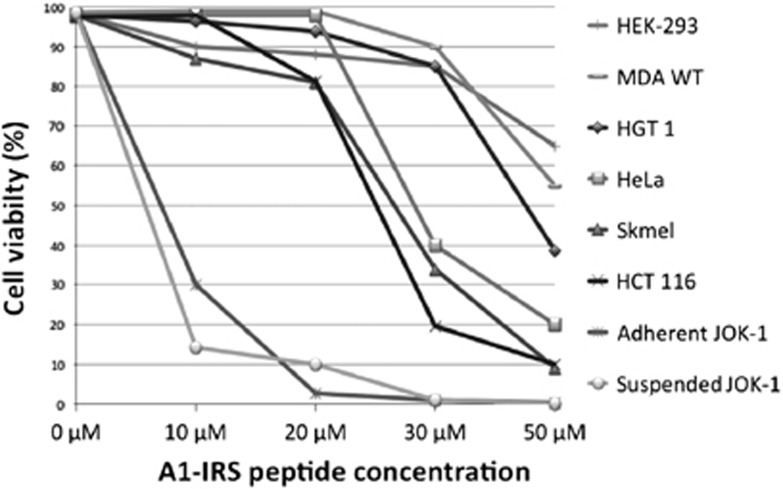
A1-IRS induces cell death in various cell lines. Cell cultures were incubated with increasing concentrations of the synthetic A1-IRS peptide for 60 min and cell death was determined as described in Figure 2b. Results are expressed as the mean of three independent experiments. HEK-293, adenovirus-transformed epithelial embryonic kidney cells; MDA, mammary gland ductal carcinoma; HGT1, gastric adenocarcinoma; HeLa, cervix adenocarcinoma; Skmel, melanoma; HCT116, colorectal carcinoma; JOK-1, chronic lymphocytic leukemia

The A1-IRS peptide showed cytotoxic activity in HeLa cells that are derived from a cervix adenocarcinoma. We therefore evaluated the cytotoxicity of A1-IRS in other cancer cell lines established from mammary gland ductal carcinoma (MDA), gastric adenocarcinoma (HGT1), cervix adenocarcinoma (HeLa), melanoma (Skmel), colorectal carcinoma (HCT116), chronic lymphocytic leukemia (JOK-1) and from adenovirus transformed epithelial embryonic kidney cells (HEK-293). Cells were incubated for 60 min with 10–50 *μ*M A1-IRS. Cell death was then assessed by double staining with Syto 13 and PI. Membrane permeabilization was efficiently induced by A1-IRS in all tested cell lines (Figure 3), but, as expected, not by the A1m-IRS variant peptide (data not shown). However, cell lines differed in sensitivity: chronic B-lymphocytic leukemia JOK-1 cell lines that grow in suspension showed the highest sensitivity, whereas the embryonic kidney cell line was only moderately affected by the peptide, with less than 40% of cell death observed at 50 *μ*M A1-IRS (Figure 3). Given that JOK-1 cells are grown in suspension culture and the other cell types are adherent culture, we tested whether culture type changes the sensitivity of the response to the peptide. In suspension culture, cells are uniformly exposed to the culture medium, whereas in adherent cultures, cells are in a monolayer and are only exposed on one side. JOK-1 cells incubated in a serum-free medium for 12 h became adherent, 10% serum was then added and they were subsequently cultured 24 h before assessing the cytotoxic activity of the peptide. As shown in Figure 3, adherent JOK-1 cells showed an A1-IRS dose–response profile similar to that of JOK-1 cells in suspension, indicating that the pattern of JOK-1 sensitivity was not due to culture conditions, but due to the intrinsic characteristics of the cell line. Given the maximum sensitivity of JOK-1 cells to A1-IRS, we conducted all subsequent experiments on this cell line.

### A1-IRS cytotoxic activity does not require active translation, but depends on eIF4E availability

**Figure 4:**
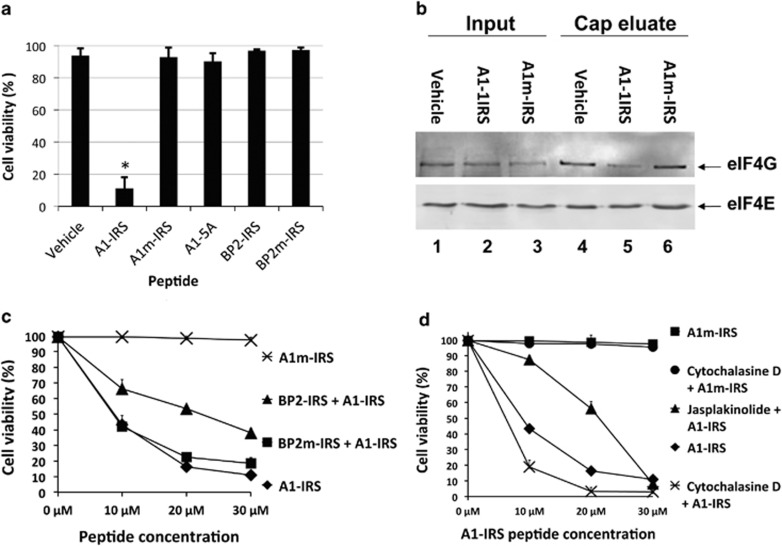
A1-IRS cytotoxicity requires eIF4E availability and causes necrosis *via* F-actin destabilization. (**a**) JOK-1 cells were incubated with 50 *μ*M of the indicated peptides and analyzed as described for HeLa cells in Figure 2b. The data are given as the mean±S.D. of triplicates, and are representative of three experiments. *Versus* group control (vehicle): * *P*<0.001. (**b**) JOK-1 cells were incubated for 1 h with A1-IRS, A1m-IRS or the vehicle (DMSO). Protein extracts were incubated with m^7^GTP beads to purify eIF4E and its associated factors; eluted proteins were analyzed by western blot with antibodies against eIF4E and eIF4G. (**c**) Increasing concentrations (from 0 to 30 *μ*M) of A1-IRS or A1m-IRS peptides were added in JOK-1 cells that were previously incubated with 200 *μ*M BP2-IRS or BP2m-IRS for 1 h. After 10 min, cell viability was determined as described in **a**. Results are representative of three independent experiments. (**d**) JOK-1 cells were pre-treated with 0.5 ng/*μ*l cytochalasin D for 1 h, or with jasplakinolide for 2 h. A1-IRS or A1m-IRS was then added at the indicated concentration and cell viability was determined after 10 min as described in (**a**). Results are representative of three independent experiments

In HeLa cells, only A1-IRS induced cell death, and not BP2-IRS (Figure 2b). We observed a similar pattern of cell viability for JOK-1 cells (Figure 4a), indicating that not only is the specific sequence of the Angel1 eIF4E-binding motif necessary to induce cell death but also it does not depend on the cell line. Although A1-IRS and BP2-IRS were both able to inhibit *in vitro* translation, they showed differences in cytotoxicity, which may possibly be due to translation repression by A1-IRS of specific messenger mRNA. If so, a general inhibitor of translation should cause cell death to occur. Preincubation of JOK-1 cells with emetine, a translation inhibitor, for 1 h induced translation arrest, as attested by the absence of ^35^S met incorporation. This translation arrest was not sufficient to induce cell death after 1 h (Supplementary Figure S4) and even after 4 h of incubation (data not shown). Emetine-pretreated cells were incubated with A1-IRS, and cell death was efficiently induced (Supplementary Figure S4). We conclude that A1-IRS induces cell death independently of translation activity.

As the cytotoxic activity of A1-IRS depends on its eIF4E-binding motif, we looked for the relationships between A1-IRS and eIF4E. Various partners of eIF4E—including Angel1, eIF4G and 4E-BP proteins—use the same binding motif and compete with each other to bind to eIF4E.^14^ When we purified eIF4E and its associated partners from cells incubated with A1-IRS, A1-IRS displaced the eIF4G-eIF4E interaction (Figure 4b, lane 5). As expected, this effect was not observed with the mutated peptide A1m-IRS that cannot bind to eIF4E (Figure 4b, lane 6). Therefore, A1-IRS can interfere with eIF4E complexes in cells.

We then tested whether eIF4E availability was a necessary prerequisite for the cytotoxic effect of A1-IRS. Cells were preincubated with a high amount of BP2-IRS (200 *μ*M) to sequester eIF4E before adding A1-IRS. Although a 1 h preincubation with BP2-IRS did not cause membrane permeabilization in JOK-1 cells in the absence of peptide addition (Figure 4a), preincubation with BP2-IRS decreased the cytotoxic effect of A1-IRS (Figure 4c). Co-incubation with the control peptide BP2m-IRS, which cannot bind to eIF4E, did not significantly affect A1-IRS activity. The cytotoxic effect of A1-IRS therefore requires eIF4E availability.

### A1-IRS causes necrosis through actin disorganization

We performed a set of experiments, summarized in Table 1, to characterize the mechanism of cell death mediated by A1-IRS. Intracellular ATP levels have been implicated both *in vitro* and *in vivo* as a determinant of the mechanism of cell death.^21^ We observed a rapid decrease in ATP levels in JOK-1 cells 10 min after addition of A1-IRS (Supplementary Figure S5). We observed by simultaneous monitoring of the release of adenylate kinase as a marker of cell membrane permeabilization (see Materials and Methods), that drop in ATP and cytolysis were concomitant (Supplementary Figure S5). Membrane blebs are typical of necrosis, they grew without retraction and reached diameters of up to several tens of micrometers, followed by plasma membrane permeabilization to PI and release of cytosolic proteins.^22^ As there are no absolute criteria to unequivocally identify necrosis, this type of cell death must be confirmed by eliminating the possibility of the occurrence of apoptosis, autophagy or other ‘atypical' cell death modalities, as established by the Nomenclature Committee on Cell Death.^23^ The caspase inhibitor ZVAD-FMK did not inhibit AI-IRS cytotoxicity, and there was no PARP, caspase 3 or caspase 8 cleavage after incubation with A1-IRS (Supplementary Figures S6 and S7), indicating that these proteins were not activated. We did not observe any nuclear fragmentation under optical (Figure 2) or electronic microscopy (data not shown). Inhibition of phospholipase C did not affect the cytotoxicity of A1-IRS (Supplementary Figure S8). We also excluded cell death due to autophagy because the inhibitor 3-methyladenine did not inhibit A1-IRS cytotoxic activity (Supplementary Figure S6) and because there were no double membrane autophagic vacuoles under electronic microscopy (data not shown). Inhibition of RIP1 by necrostatin-1 did not inhibit A1-IRS activity, thereby excluding necroptosis (Supplementary Figure S9).^24^ We also looked for mitochondrial perturbation and did not observe any cytochrome C redistribution (Supplementary Figure S10) or ROS increase (Supplementary Figure S11) after incubation with A1-IRS, and mitochondria appeared normal when observed under electronic microscopy (data not shown). The cytosolic concentration of calcium did not increase (Supplementary Figure S12) and cathepsin inhibition (Supplementary Figure S13) did not impede A1-IRS activity. Taken together, these results indicate that we did not observe the properties of any specific cell death modality. We therefore concluded that the observed cell death is a necrotic cell death characterized by massive cell blebbing, leading to an early loss of integrity of the plasma membrane and a drop in ATP levels.

Blebs generally appear when the actin links between the plasma membrane and the underlying cytoskeleton are weakened.^25^ Because the actin cytoskeleton network is also implicated in cell death regulation in a wide variety of cells,^26^ we tested whether actin dynamics have a role in the cytotoxicity of A1-IRS. We preincubated cells with cytochalasin D or jasplakinolide, which respectively, prevent actin polymerization and inhibit depolymerization of actin microfilaments, prior to the addition of A1-IRS. As expected, cytochalasin D treatment alone had no effect on PI permeabilization (Figure 4d), but pre-incubation with cytochalasin D potentialized A1-IRS cytotoxicity as shown by a dose–response experiment (Figure 4d). Conversely, jasplakinolide had the opposite effect, resulting in a decreased efficiency of A1-IRS to mediate cell death (Figure 4d). To investigate the cellular distribution of actin, we performed immunofluorescence experiments on HeLa cells because our attempts to use this technique for JOK-1 cells were unsuccessful. We observed a dramatic disorganization of F-actin microfilaments in cells incubated with A1-IRS (Supplementary Figure S14). Examination of protein levels using western blotting showed that B-actin was unaffected by A1-IRS treatment in both JOK-1 (Supplementary Fig. S7) and HeLa cells (not shown). Taken together, these experiments demonstrate that actin network destabilization is a necessary step for the cytotoxic effect of A1-IRS.

## Discussion

Here, we generated an original peptide, A1-IRS, derived from Angel1, a partner of eIF4E that we recently identified.^14^ The A1-IRS peptide interacts with eIF4E, inhibits *in vitro* translation and, surprisingly, provokes rapid and massive cell death in a wide variety of cancer cell lines. The induced necrosis is characterized by a drop in ATP levels, with F-actin network injury being a key step to the rupture of the plasma membrane. This cytotoxicity was not due to the general physicochemical properties of A1-IRS, as this non-amphipathic peptide displays similar charge and hydrophobicity profiles as its inactive counterpart A1m-IRS (data not shown).

Through its binding to eIF4E, A1-IRS competes eIF4G (Figure 4b) and is able to inhibit translation (Figure 1b and Supplementary Figure S2). However, in the absence of translation, rapid plasma membrane permeabilization still occurred upon incubation with A1-IRS, indicating that translation initiation activity of eIF4E is not the cause of cell death induced by the peptide. Although we cannot formally exclude the possibility that the partial inhibition of translation induced by A1-IRS in living cells (Supplementary Figure S2) is an essential prerequisite for cell death, the rapid change in cell permeability seems to happen too quickly to be due to inhibition of protein synthesis.

As BP2-IRS did not cause cell death, the eIF4E-binding property of a peptide is not sufficient to induce cell death. Therefore, it remains possible that A1-IRS is bound to molecular targets other than eIF4E to mediate cell death. However, BP2-IRS preincubation decreased the cytotoxic activity of A1-IRS (Figure 4c), suggesting that eIF4E must be available for A1-IRS to produce its cytotoxic effect. Furthermore, in addition to A1m-IRS, two other point mutations expected to decrease binding to eIF4E showed a reduced capacity to inhibit *in vitro* translation and mediate cell death (data not shown). Thus, eIF4E binding capacity and cytotoxic activity are tightly linked.

Earlier studies have established a possible link between eIF4E and actin dynamics. eIF4E associates with two different actin networks in dendrites; treatment with brain-derived neurotrophic factor increases the proportion of eIF4E to levels similar to that located in dendritic spine heads consisting of a highly branched network of filaments.^27^ Furthermore, actin and eIF4E have been co-purified by m^7^GTP-Sepharose chromatography from migrating cell extracts.^28^ eIF4E has also been found associated with actin-rich pseudopodia in six metastatic tumor cell lines, eIF4E knockdown induced reduced F-actin density and slower loss of F-actin during treatment by the depolymerizing drug latrunculin A.^29^ Importantly, expression of a constitutively hypophosphorylated 4E-BP1 sequesters eIF4E and inhibits IGF-I-stimulated F-actin reorganization and lamellipodia formation.^30^ All of these findings are consistent with our result whereby F-actin was reorganized during incubation with A1-IRS, suggesting a functional link between eIF4E and F-actin dynamics.

An eIF4E-interacting peptide derived from BP2 fused to a penetratin sequence was previously shown to induce cell death, which shows characteristics of apoptosis and observed only with 72 h serum-starved cells.^12^ The small molecule 4EGI-1 that also inhibits eIF4E–eIF4G interaction mediates a TRAIL-mediated apoptosis through c-FLIP downregulation and DR5 induction.^13, 31^ BP2 peptide—penetratin, 4EGI-1 and A1-IRS primarily target eIF4E and induce cell deaths independently of translation inhibition. These results emphasize the need to further investigate the role of eIF4E in the control mechanisms of cell survival.

Tumor-specific peptide agents hold great promise for clinical applications in cancer diagnosis and therapy. Our results show that A1-IRS may be a candidate pharmacophore to induce rapid cell death. The cell lines that we tested differed in sensitivity (Figure 3). Therefore, this cytotoxic peptide may have clinical utility and should be improved by selective targeting. The high sensitivity of JOK-1, a cell line established from a patient with chronic lymphocytic leukemia, suggests that A1-IRS may be an interesting tool for therapeutic strategies in this type of cancer. As antibody fusion could be used as a specific tool for cell targeting, work is currently underway to evaluate whether an A1-IRS-antibody fusion is a useful approach.

## Materials and Methods

### Peptides and other reagents

Peptides were produced and purified by HPLC (purity >98%), before lyophilization (GeneCust, Dudelange, Luxembourg). Peptides were dissolved at 50 mM in 99.8% DMSO (Sigma, Saint-Quentin Fallavier, France), and then diluted to different concentrations in water or in culture medium for cell-based experiments. The final concentration of DMSO in cultures was always less than 0.4% (v/v). Sequences of the peptides used are listed in Figure 1a. A translation grade ^35^S-methionine was used (EasyTag Methionine L-[^35^S], PerkinElmer, Waltham, MA, USA).^32^

### Analysis of *in vitro* interactions between YFP-A1 and GST-eIF4E

After production and purification, GST-eIF4E recombinant protein^17^ was dialyzed overnight in buffer A (50 mM HEPES pH 7.7, 150 mM KCl, 1 mM EDTA, 5% glycerol). Then, 1 *μ*g of GST-eIF4E was incubated for 1 h with 25 *μ*l of GSH-Sepharose beads in buffer A. After washing, the beads were incubated for 1 h in buffer A containing 1 mg/ml of bovine serum albumin, 0.5% Igepal with 10 *μ*l rabbit reticulocyte lysate (TnT T7 Coupled Reticulocyte Lysate System, Promega, Charbonnières, France) that was programmed with YFP, YFP-A1 or YFP-A1m PCR DNA (all without the sequence coding for the penetratin IRS domain, see text) in presence of [^35^S] methionine. After extensive washing, the beads were boiled in Laemmli buffer and analyzed by autoradiography on a Typhoon Trio imager (Amersham Pharmacia Biotech, Uppsala, Sweden).

### Translation assay in rabbit reticulocyte lysate

Capped mRNAs coding for Renilla luciferase (Luc R) were produced by a T7 Message Machine Kit system (Ambion, Life Technologies, Saint Aubin, France) from pGb-Eg2-410Δ2-hxG-A65 *Eco*RV-linearized vector.^33^ RNA was then purified by phenol-chloroform extraction and controlled by gel electrophoresis. *In vitro* translation was carried out in the Flexi-rabbit reticulocyte system (RRL, Promega), supplemented with 20 mM amino acids (Promega), 1 U/*μ*l RNase inhibitor (Ambion), potassium chloride (KCl), and 5 fmol mRNA per reaction. Renilla activity from translation reaction was measured using the Renilla Luciferase Assay System (Promega) on a Tristar LB941 luminometer (Berthold, Thoiry, France).

### Cell culture

JOK-1 cells were grown in complete RPMI 1640 medium with glutaMAX (Gibco BRL, Life Technologies) supplemented with 10% heat-inactivated fetal calf serum, 1 unit/ml penicillin, 1 *μ*g/ml streptomycin and 1% L-glutamine, (Gibco BRL). For adherent cell cultures, cells were grown in complete Dulbecco's modified Eagle medium (Gibco BRL) with 10% heat-inactivated fetal calf serum, 1 unit/ml penicillin, 1 *μ*g/ml streptomycin and 1% L-glutamine (Gibco BRL). All cell lines were grown at 37 °C in a 5% CO_2_ humidified atmosphere.

### Time-lapse microscopy

Cells were loaded in black 96 well plates with glass bottoms and 40 000 cells per well. Before peptide treatment, 5 *μ*M Syto 13 (Molecular Probes, Gibco BRL) and 1 mg/ml propidium iodide (PI) (Sigma) were added to the culture medium.^34^ FM-464 (Molecular Probes, Gibco BRL) was used at 1 *μ*M before recording Supplementary Movie 1. Fluorescence was followed at various time intervals with a Zeiss Observer Z1 (Zeiss, Marly Le Roi, France) or a Leica TCS SP5 microscope (Leica, Nanterre, France). When indicated, cell morphology was followed by DIC and phase contrast microscopy.

### Immunofluorescence microscopy

HeLa cells (2 × 10^4^) were plated on 8-well Lab-Tek II Chamber Slides and grown overnight. After incubation with peptides or the vehicle (DMSO), cultures were washed three times with PBS, treated with BD Cytofix/Cytoperm kit (4% paraformaldehyde with 0.1% saponin and 3% fetal calf serum (Cat No.554714 BD Biosciences, Le Pont de Claix, France) at 4 °C for 20 min. Fixed and permeabilized cells were incubated with primary antibody diluted in BD Cytoperm/Wash solution. After overnight incubation at 4 °C, cells were washed and incubated with the appropriate secondary antibody at room temperature for 1 h. Cells were washed three times (10 min each) in BD Perm/Wash Buffer and stained with 1 *μ*g/ml Hoechst 33342 (Sigma) in BD Perm/Wash Buffer for 10 min. After two further washes, the slides were covered with a cover slip, using Fluoprep (BioMérieux, Lyon, France) as a mounting medium and sealed with clear nail polish. Images were collected on a Zeiss fluorescence microscope using a × 63 oil objective. Polyclonal rabbit antibodies raised against the A1 peptide RRKYGRDFLLC (P16; 1 : 500) were used to localize the A1-IRS peptide. No signal was detected after incubating cells with A1m-IRS, presumably because of the failure of the antibody to recognize the mutated sequence. Secondary antibodies used were Cy 3-conjugated AffiniPure Donkey anti-mouse IgG (Jackson ImmunoResearch laboratories, West Grove, PA, USA) at a concentration of 1 : 500, or anti-rabbit conjugated to Alexa Fluor 488 (Molecular Probes) at a 1 : 500 dilution. F-Actin was visualized using a phalloidin-rhodamine conjugate (Molecular Probes), at a final concentration of 0.3 *μ*M. Before observation under microscope, HeLa cells were washed in BD Perm/Wash Buffer with 1% bovine serum albumin.

### Detection of ToxiLight AK and determination of ATP levels

JOK-1 cells were diluted to 30 000 cells per well in 100 *μ*l of culture medium, and were plated on 96-well plates. Each peptide was diluted in cell medium to reach the indicated concentration. After incubation with the peptide, analysis of adenylate kinase release was measured using the ToxiLight BioAssay Kit (Lonza, Levallois Perret, France), and ATP levels were determined with the CellTiter-Glo Luminescent Cell Viability Assay (Promega) according to the manufacturer's instructions in a Tristar LB941 luminometer (Berthold).

## Figures and Tables

**Table 1:** List of the experiments conducted and the drugs used to characterize the cytotoxicity of A1-IRS

**Analysis**	**Experiment**	**Figure**	**Result**
Microscopy	PI staining detection	2	+
Luminescence	Drop in ATP levels detection	S5	+
Microscopy	Nuclear fragmentation detection	2	−
Western blot	Caspase 3 cleavage detection	S7	−
Western blot	Caspase 8 cleavage detection	S7	−
Western blot	PARP cleavage detection	S7	−
Western blot	MCL1 cleavage detection	S7	−
Western blot	Cytochrome C release	S10	−
Microscopy	ROS increase	S11	−
Microscopy	Calcium increase with Fluo4-AM probe	S12	−

**Reagent**	**Target**	**Figure**	**Cytotoxicity of A1-IRS**
ZVAD-fmk	Caspases	S6	−
U73122	Phospholipase C	S8	−
3-methyladenine	Autophagic cell death	S6	−
Necrostatin-1	Rip1	S9	−
CA-074Me	Cathepsin	S13	−
Emetine	Translation	S4	−
4E-BP2 peptide	eIF4E	4c	Slowed
Cytochalasin D	Actin F destabilization	4d	Accelerated
Jasplakinolide	Actin F stabilization	4d	Slowed

## Abbreviations

eIF: eukaryotic initiation factor
ATP: adenosine triphosphate
F-actin: filamentous actin
4E-BP: 4E-binding protein
mTOR: mammalian target of rapamycin
A1: Angel1
YFP: yellow fluorescent protein
PI: propidium iodide
DMSO: dimethyl sulfoxide
DIC: differential interference contrast
PARP: poly ADP ribose polymerase
RIP: receptor interacting protein
ROS: reactive oxygen species
m^7^GTP: 7-methylguanosine 5'-triphosphate
IGF-I: insulin-like growth factor-1
c-FLIP: cellular FLICE (FADD-like IL-1*β*-converting enzyme)-inhibitory protein
TRAIL: tumor necrosis factor-related apoptosis-inducing ligand
DR: death receptor
MCL1: myeloid cell leukemia 1
