# Supplementary material for: An eIF4E-interacting peptide induces cell death in cancer cell lines
Source: Cell Death Dis. 2014 Oct 30;5(10):e1500–. doi: 10.1038/cddis.2014.457 (PMC4237268; doi:10.1038/cddis.2014.457)
Supplement: Supplementary Figures and Legends [file cddis2014457x1.pdf]

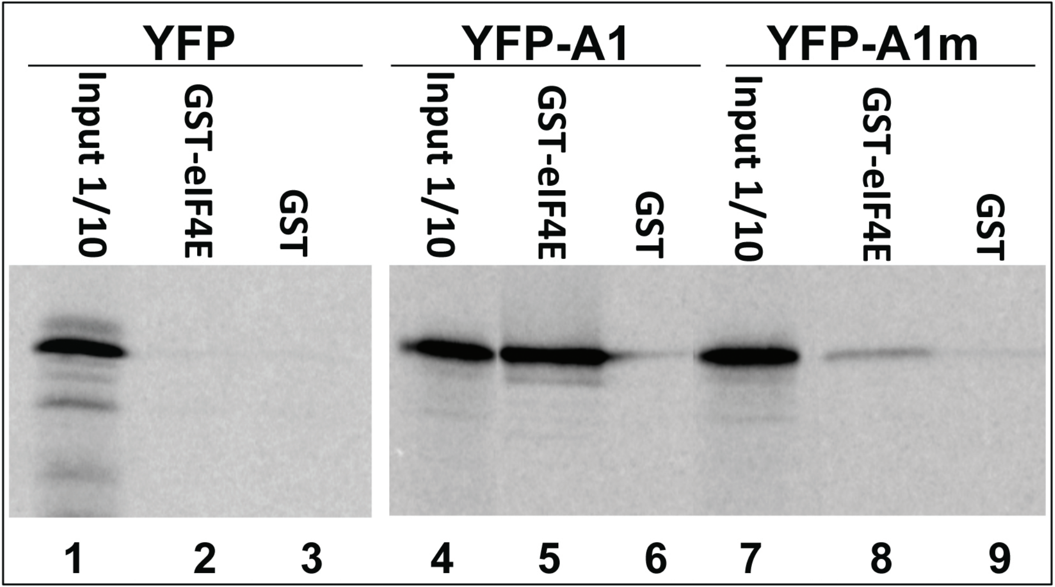

Masse et al., Fig. S1

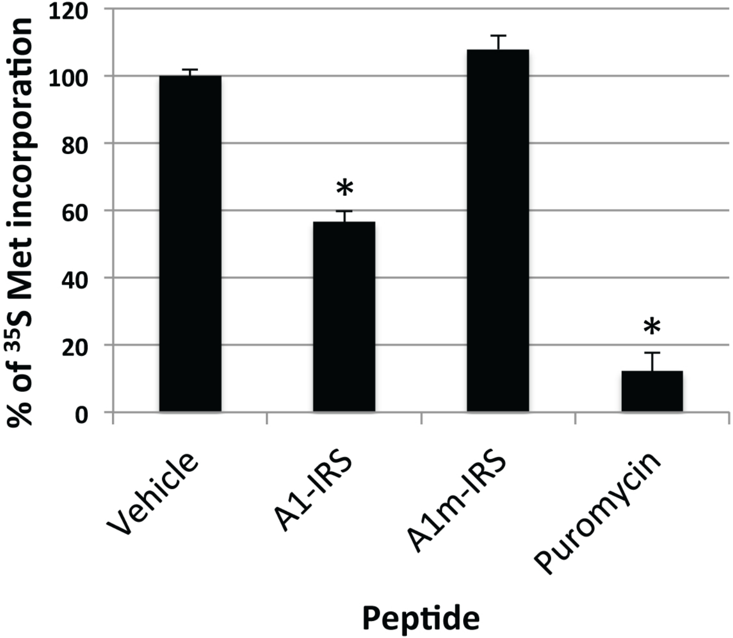

Masse et al., Fig. S2

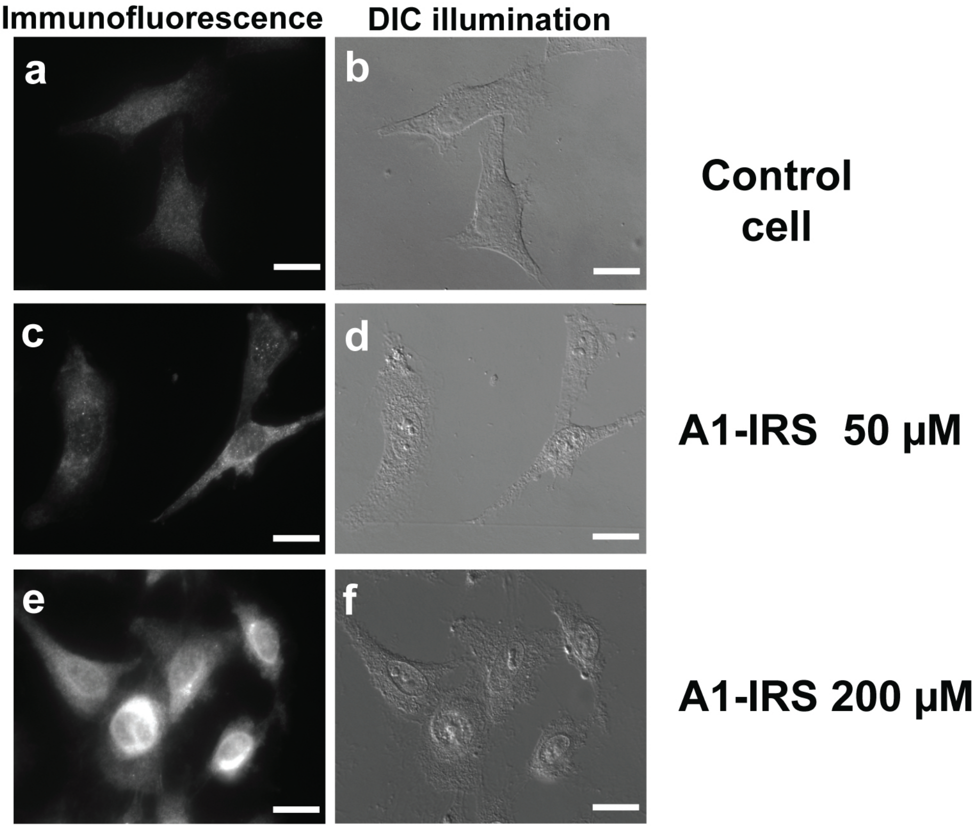

Masse et al., Fig. S3

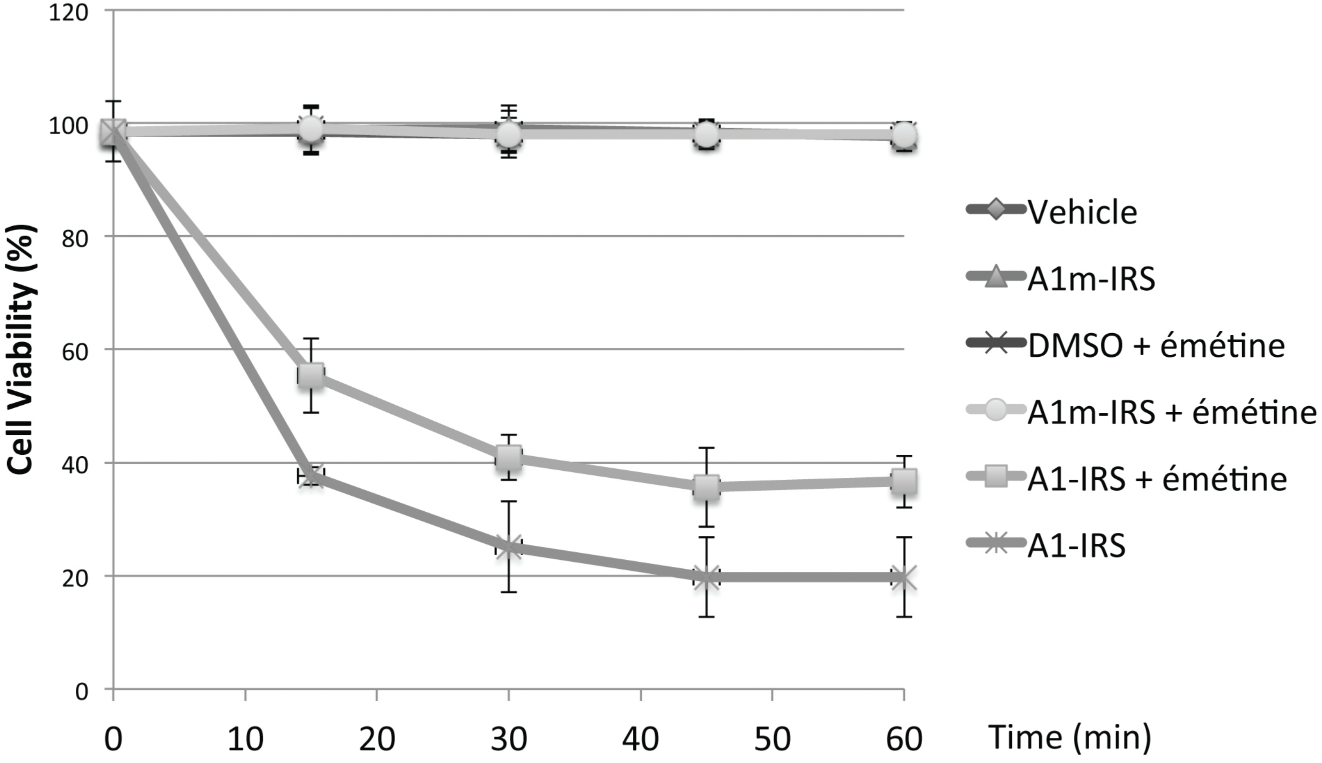

Masse et al., Fig. S4

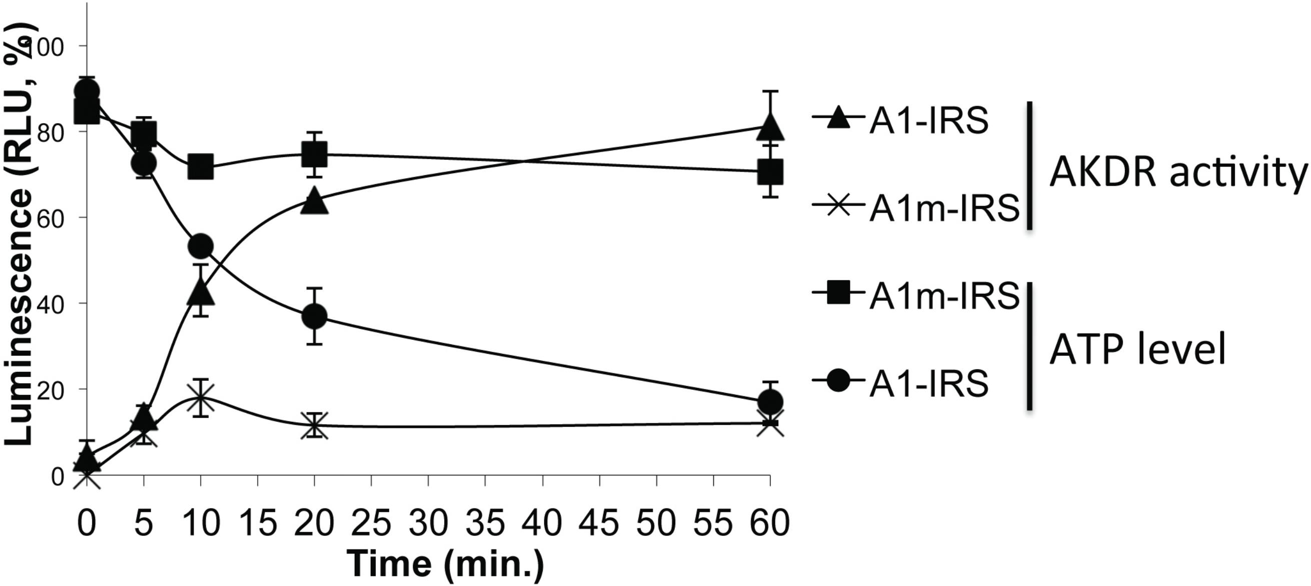

Masse et al., Fig. S5

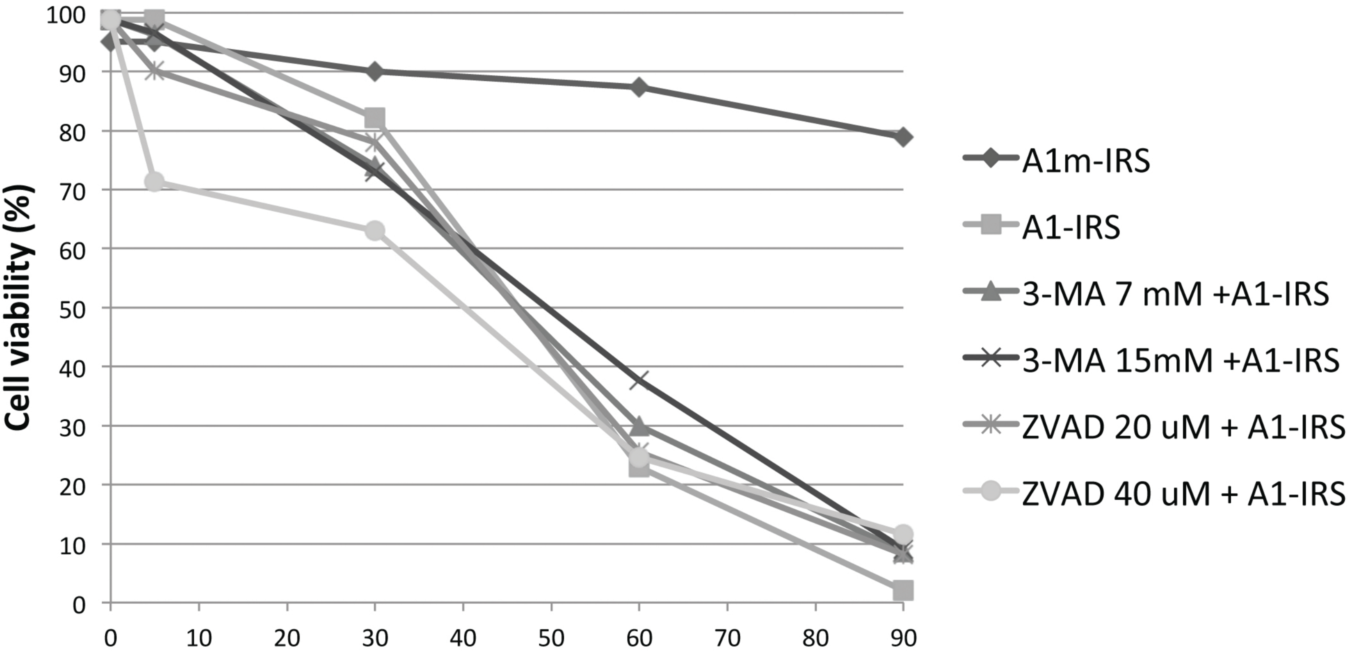

Masse et al., Fig. S6

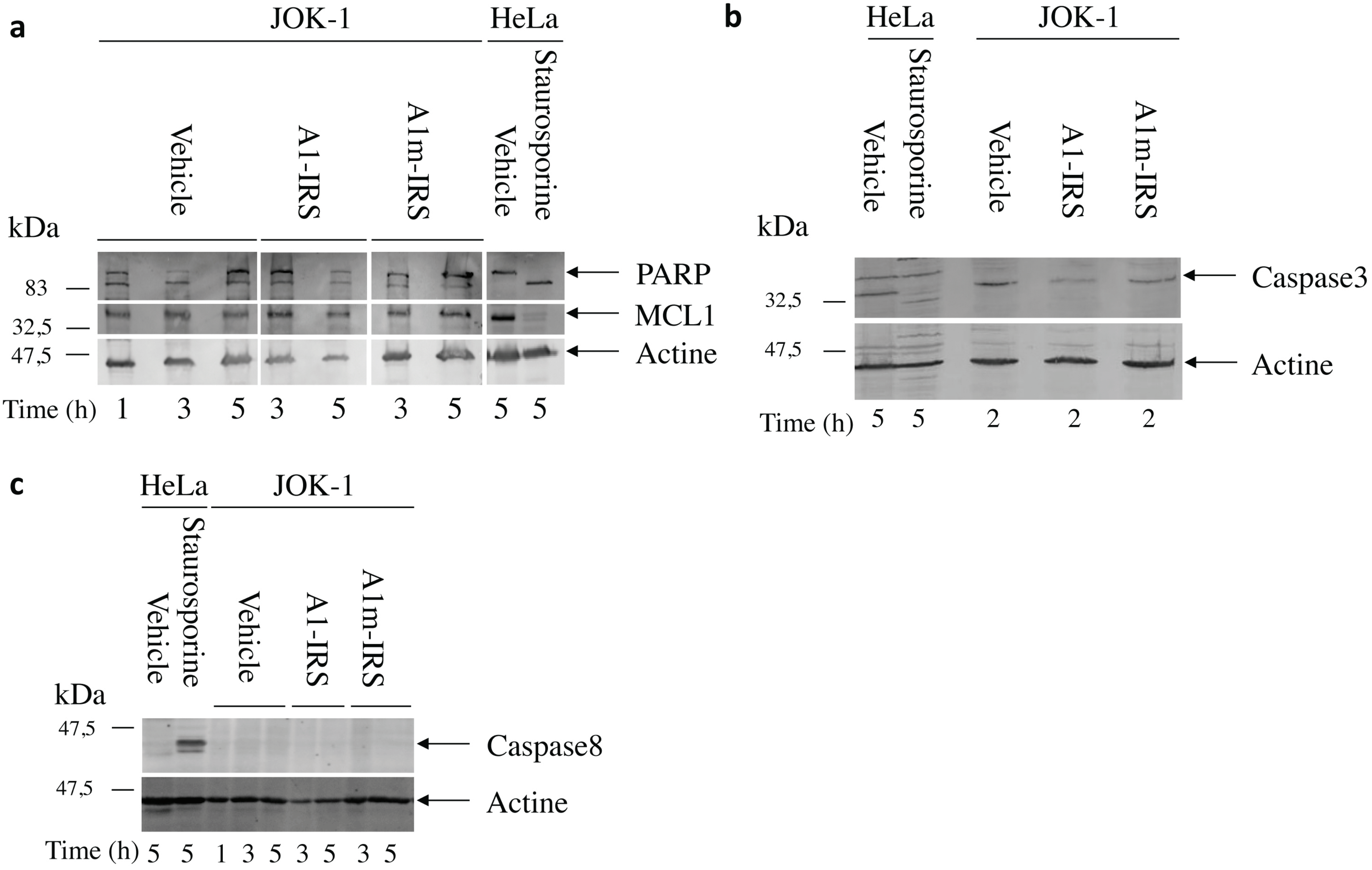

Masse et al., Fig. S7

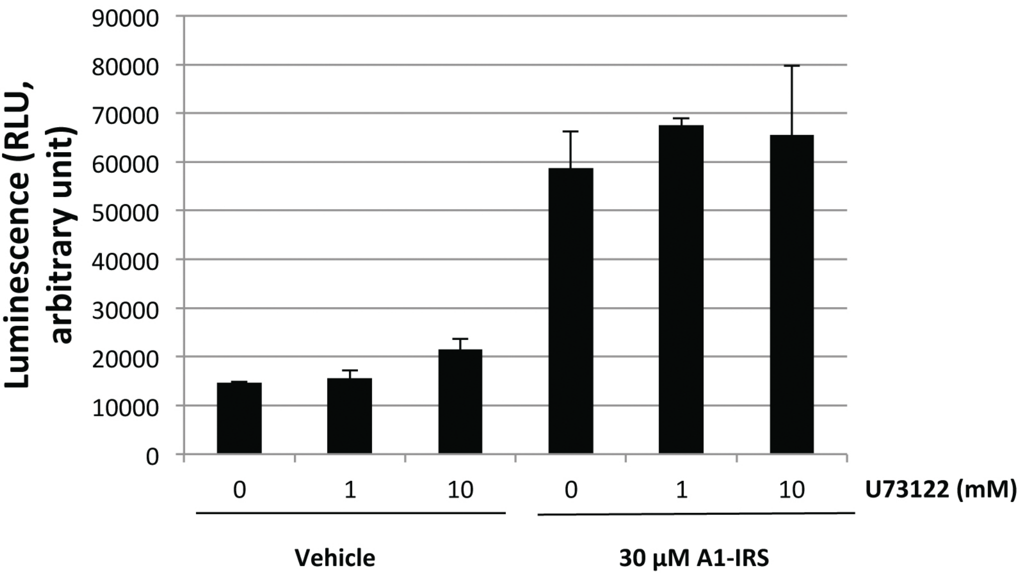

Masse et al., Fig. S8

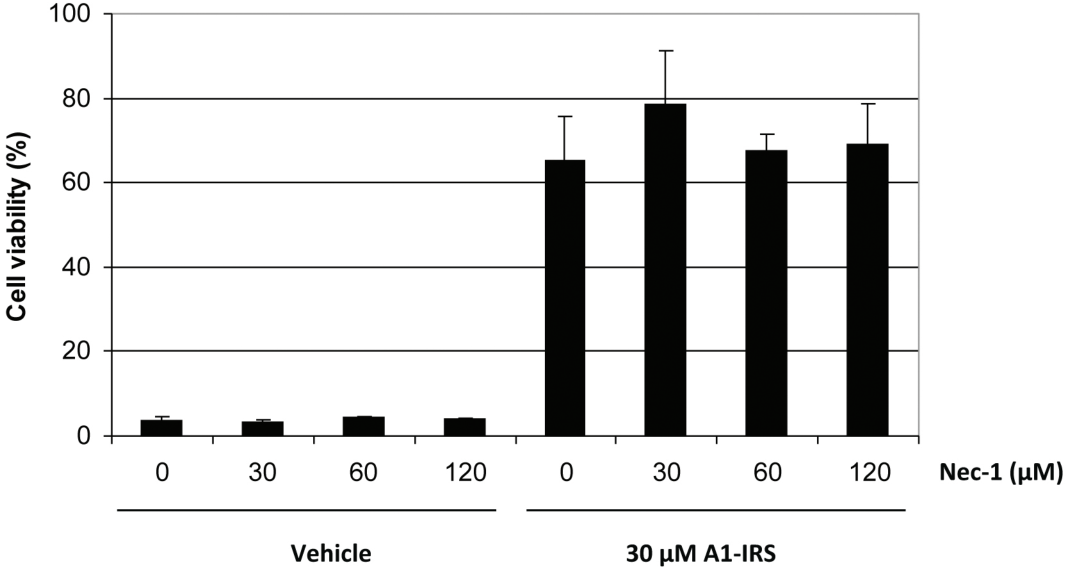

Masse et al., Fig. S9

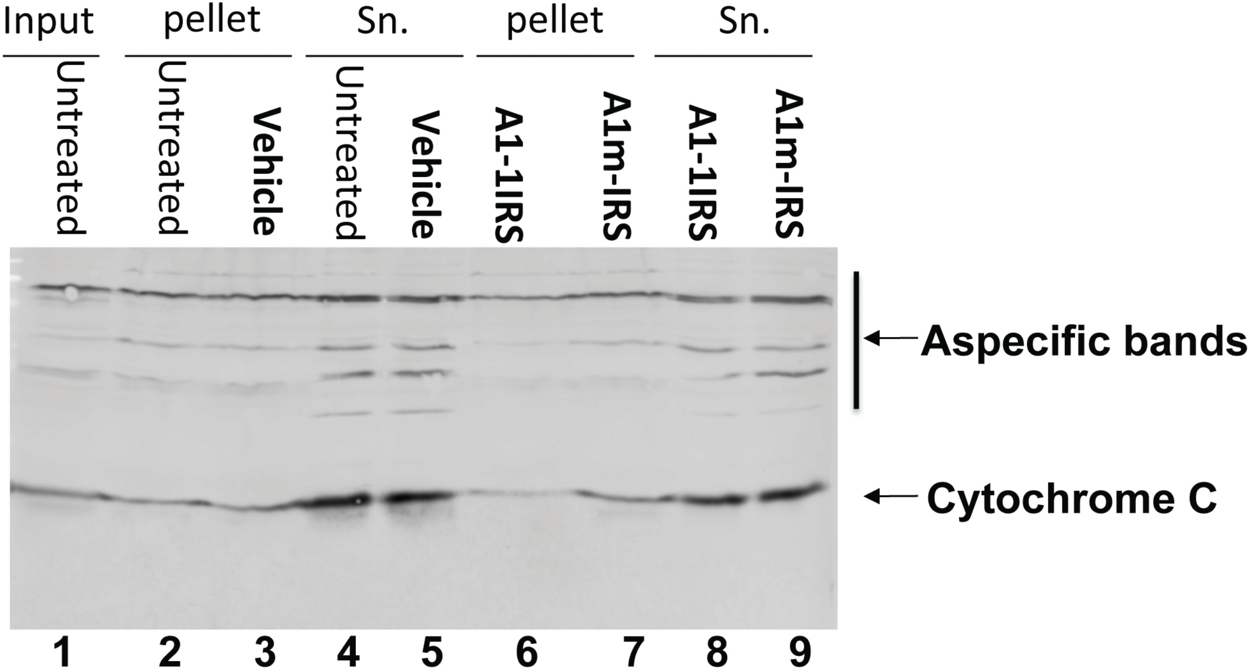

Masse et al., Fig. S10

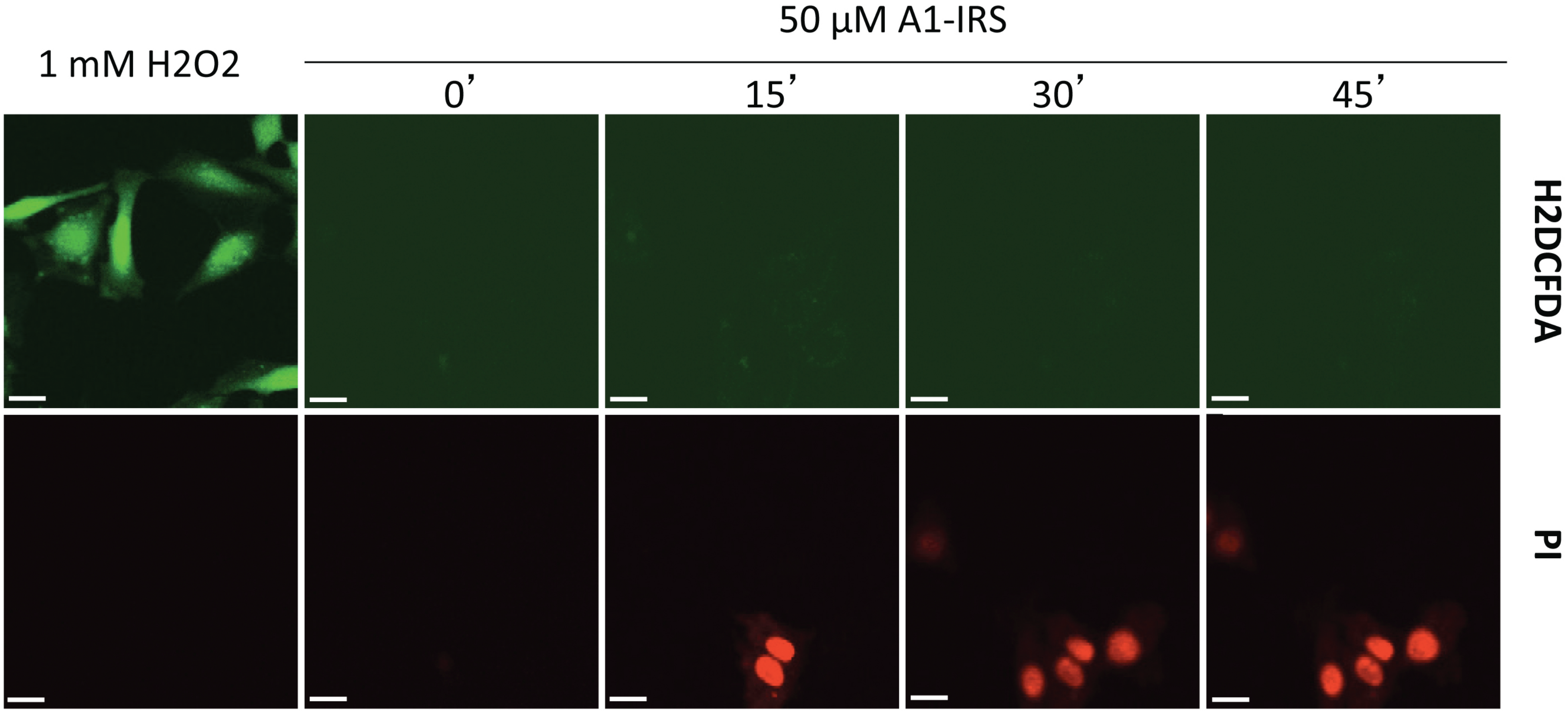

Masse et al., Fig. S11

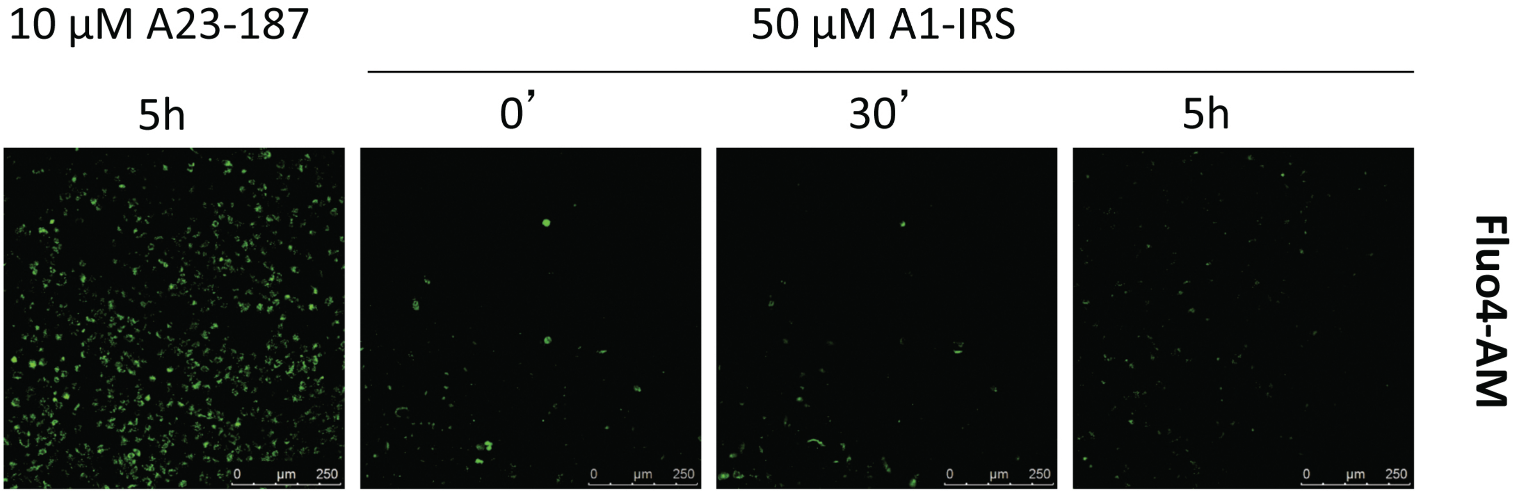

Masse et al., Fig. S12

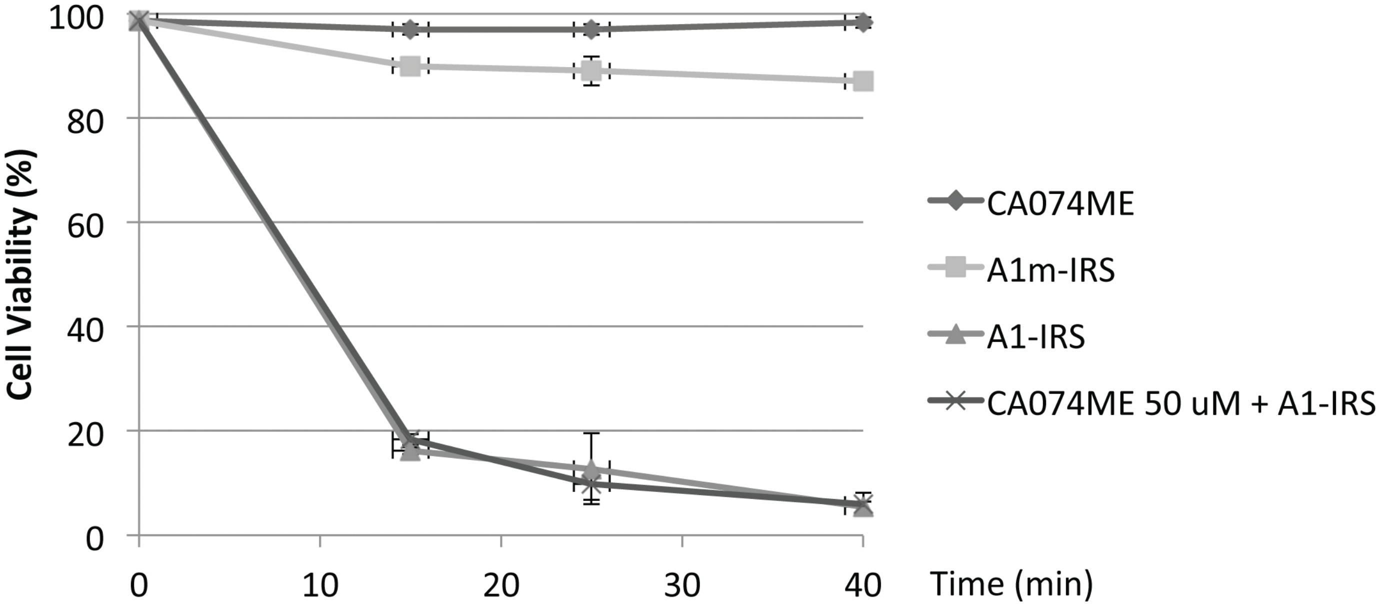

Masse et al., Fig. S13

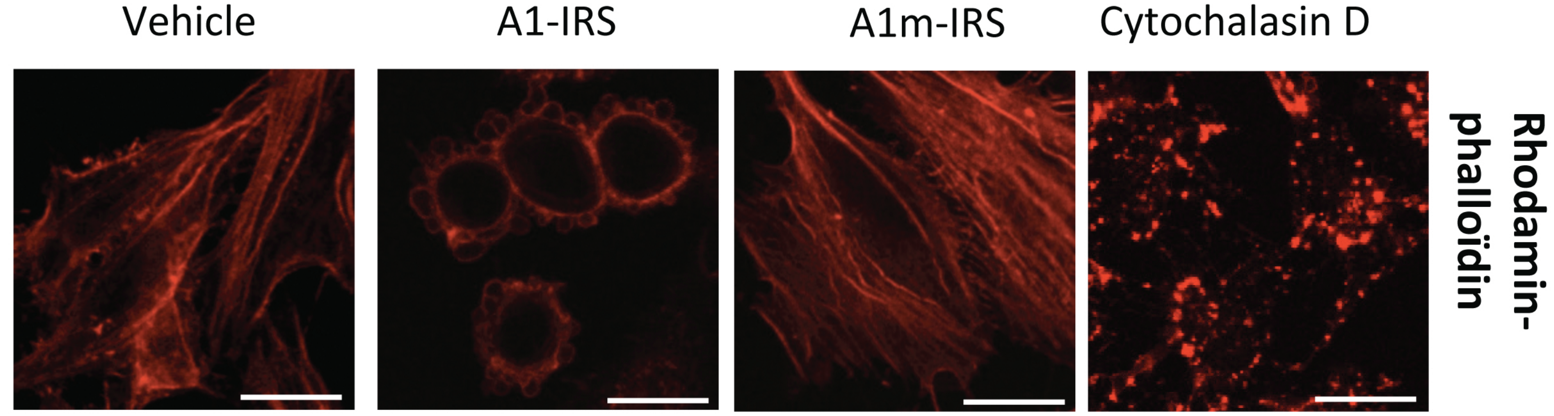

Masse et al., Fig. S14

Supplementary Figure Legends

Supplementary Figure 1. A 12 amino acid peptide derived from Angel1 binds to eIF4E *in vitro*. ^{35}S-methionine-labeled yellow fluorescent protein (YFP) fused to the A1 peptide or its A1m variant (see text for details) were produced in rabbit reticulocyte lysates. Translation products were loaded on a m^{7}GTP column pre-loaded with GST-eIF4E or GST as controls. After washing, proteins were eluted in Laemmli-sample buffer, resolved by SDS-PAGE and analyzed by autoradiography. Inputs that represent 10% of the volume used in the experiment were loaded in parallel (lanes 1, 4, 7).

Supplementary Figure 2. A1-IRS peptide inhibits translation in living cells. Pulse labeling of HeLa cells in vivo with ^{35}S-methionine showed a 2-fold decrease in the rate of protein synthesis after treatment with A1-IRS. HeLa cells were treated 30 min with 50 uM A1-IRS or A1m-IRS, or with 2 ug/ml of the translational inhibitor puromycin as control. Cells were then cultured in presence of ^{35}S-methionine at the final concentration of 5 µCi/ml during 20 min. ^{35}S-methionine incorporation into proteins was measured on duplicate aliquots of cell extracts after 10% TCA precipitation on Whatman 3M filters as described previously and counting in a scintillation counter in the presence of Optiphase Supermix scintillation liquid. [32] The data are given as the mean ± SD of triplicates, and are representative of two experiments. The average incorporation of the control (vehicle) was set to 100%. Versus group control (vehicle) : *; P<0,001.

Supplementary Figure 3 A1-IRS localizes intracellularly. HeLa cells were incubated with 50 or 200 µM of A1-IRS for 1 h at 37°C and then immunostained with antibodies directed against the A1 peptide (images a, c and e). Cellular structures were observed with DIC illumination (images b, d and f). Scale bar = 20 µm.

Supplementary Figure 4. A1-IRS induces cell death independently of translation activity. JOK-1 cells were incubated with or without 110 µM of emetine for 1 hour before adding

30 μM A1-IRS, A1m-IRS or the DMSO (vehicle). Cell viability was determined with PI and Syto 13 as described in Fig.2.

Supplementary Figure 5 A1-IRS causes plasma membrane permeabilization with a concomitant drop in ATP levels. Samples from JOK-1 cells were treated in parallel to determine ATP levels in cells and adenylate kinase activity in the culture medium after incubation during the indicated time with 10 μM A1-IRS or the variant peptide A1m-IRS. Values obtained for ATP in untreated cells were arbitrarily set to 100%.

Supplementary Figure 6: The autophagy inhibitor 3-MA or the caspase inhibitor ZVAD-FMK did not inhibit AI-IRS cytotoxicity. JOK-1 cells were pre-incubated with 7 or 15 mM of 3-Methyladenine (3-MA) for 30 minutes, or with 20 or 40 μM of Z-VAD-FMK for 1 hour before adding 50 μM A1-IRS or A1m-IRS (where indicated). Cell viability was determined as described in Fig.2.

Supplementary Figure 7: PARP, Mcl1, Caspase 3, Caspase 8, were not cleaved after incubation with A1-IRS. JOK-1 cells were incubated for 1, 2, 3 or 5 hours (as indicated below the figure) with 50 μM A1-IRS, A1m-IRS or DMSO (vehicle). Protein extracts were analyzed by Western-blot with antibodies against PARP (PARP-1/2 (H-250) : sc 750, Santa Cruz Biotechnology), Mcl1 (MCL1(s-19) : sc-819, Santa Cruz Biotechnology), Caspase 3 (Caspase 3 (4703), Calbiochem), Caspase 8 (Cleaved caspase 8 (Asp384), Calbiochem), and actin (Sigma Aldrich). As a positive control for the induction of apoptosis, cell extracts from HeLa cells incubated with 50 nM staurosporin were loaded in parallel.

Supplementary Figure 8: Phospholipase C inhibitor did not inhibit AI-IRS cytotoxicity. JOK-1 cells were incubated with increasing concentrations of Phospholipase C inhibitor (U73122, Sigma Aldrich) in presence 30 μM A1-IRS or DMSO (vehicle). After 1 hour of incubation, adenylate kinase release was measured as described in Materials and Methods.

Supplementary Figure 9: The necroptosis inhibitor Nec-1 did not inhibit AI-IRS cytotoxicity. JOK-1 cells (80 000 cells) were incubated with different concentrations of Necrostatin-1 (Nec-1, Sigma Aldrich) in presence of 30 μM of A1-IRS or DMSO (vehicle). After 4 hours of incubation at 37°C, cell viability was determined as described in Fig.2.

Supplementary Figure 10: Cytochrom C distribution did not vary after A1-IRS incubation. JOK-1 cells incubated for 90 minutes with 50 μM of A1-IRS, A1m-IRS or the vehicle (DMSO), were separated into soluble (cytoplasm; supernatant (Sn.)) and insoluble (mitochondria, nuclei and other organelles; pellet) fractions. 20% of the volume of the supernatant fraction and 2% of the insoluble fraction were loaded in parallel on SDS-PAGE. Western blots performed with anti-Cytochrome-C antibodies (Sigma Aldrich) showed that cytochrom C from cells incubated with A1-IRS does not increase in the cytoplasm fraction.

Supplementary Figure 11: No ROS production was observed after A1-IRS incubation. HeLa cells were first incubated with 5 μM of cell-permeant 2',7'-dichlorodihydrofluorescein diacetate (H2DCFDA, Molecular Probes®). After 20 minutes at 37°C, 50 μM of A1-IRS or 0.1 mM H2O2 for positive control and 1 mg/ml of propidium iodide was added. Cells were visualized using a laser scanning confocal microscope (Leica Microsystems Heidelberg GmbH, Mannheim, Germany).

Supplementary Figure 12: A1-IRS did not act to increase the concentration of calcium in HeLa cells. HeLa cells were treated with 5 μM of Fluo-4 AM probe (Molecular Probes), before adding 50 μM of A1-IRS or 10 μM of the calcium ionophore A23187 for positive control. Fluorescence microscopy was performed using a laser scanning confocal microscope (Leica Microsystems Heidelberg GmbH, Mannheim, Germany).

Supplementary Figure 13: Cathepsin B inhibitor did not inhibit AI-IRS cytotoxicity.

JOK-1 cells were incubated or not with 50 µM of CA074ME for 3 hour before adding 50 µM A1-IRS, A1m-IRS or DMSO (vehicle). Cell viability was determined as described in Fig.2.

Supplementary Figure 14: A1-IRS destabilizes F-actin in HeLa cells. HeLa cells were incubated with 50 µM of A1-IRS or A1m-IRS, 1 ng/µl Cytochalasin D (Sigma) or DMSO (vehicle). After 1 hour at 37°C, cells were fixed and stained with Rhodamin-phalloïdin to observe F-actin (shown in red) as described in Materials and Methods. Scale bar = 20 µm.

Supplementary Movie 1: A1-IRS causes cell blebbing that precedes membrane permeabilization. HeLa cells were stained with FM-464 to visualize the plasma membrane, and with Syto 13 and propidium iodide as described in Figure 2. The recording begins 2 min after adding A1-IRS in the cell culture medium and covers 26 min. Images were acquired at 3 sec intervals.
